# Cost-Benefit Analysis of Interpersonal Therapy and Fluoxetine for Treating Depression and PTSD in Primary Care Settings in Kenya

**DOI:** 10.21203/rs.3.rs-6977800/v1

**Published:** 2025-07-28

**Authors:** Easter Olwanda, Daniel Mwai, Muthoni Mathai, Rachel Burger, Linnet Ongeri, David Bukusi, Anne Mbwayo, Grace Rota, Ammon Otieno, Raymond Rota, Susan Meffert, James G. Kahn

**Affiliations:** Futures Health Economics and Metrics Limited; University of Nairobi; University of Nairobi; University of California, San Francisco (UCSF) - Bixby Center for Global Reproductive Health; Kenya Medical Research Institute (KEMRI); University of Nairobi; University of Nairobi; University of Nairobi; University of Nairobi; University of Nairobi; University of California, San Francisco (UCSF) - Bixby Center for Global Reproductive Health; UCSF – Philip R. Lee Institute for Health Policy Studies and IGHS

**Keywords:** Depression, PTSD, Interpersonal Therapy, Fluoxetine, Benefits: Cost ratio, Kenya

## Abstract

**Background:**

Kenya faces a significant mental health crisis, with 1.9 million reported cases of depression and 10.6% prevalence of post-traumatic stress disorder (PTSD). The economic burden of mental health conditions was 62.2 billion Kenyan shillings in 2021, accounting for 0.6% of GDP. This study performed a cost-benefit analysis (CBA) of Interpersonal Psychotherapy (IPT) and fluoxetine (FLX) for treating depression and PTSD in a primary care setting.

**Methods:**

The SMART-DAPPER project in western Kenya (Kisumu County Referral Hospital) employed a Sequential, Multiple Assignment Randomized Trial design to train non-specialist providers in administering IPT and FLX for adult depression and PTSD. A cost-benefit analysis (CBA) compared intervention costs with income gains from increased productivity, using micro-costing for treatment expenses and the World Bank’s Living Standards Measurement Study to assess productivity. The benefit-cost ratio was calculated over one and ten years with annual relapse rates of 10%, 25%, and 50%). This study adhered to CONSORT guidelines.

**Results:**

The study enrolled 1,918 participants: 986 received IPT and 932 received FLX. Remission was achieved after the first round of therapy by 782 and 798, respectively. IPT averaged 11.5 sessions of 60 minutes, costing in total 5,050 KES ($42.79); FLX averaged 5.3 sessions of 20 minutes, costing 2,511 KES ($21.28), including the medication. Both treatments increased income, with IPT participants gaining 16,242 KES and FLX participants 13,239 KES in the first year. Benefit-cost ratios were 3.2:1 for IPT and 5.3:1 for FLX. Over ten years, FLX showed higher CBA ratios (10 to 31:1) than IPT (6 to 18:1).

**Conclusion:**

This study found productivity gains greater than primary care-based treatment costs for IPT and FLX for depression and PTSD in Kenya. FLX demonstrated a more favorable benefit-to-cost ratio. Future research should address the capacity of government health care to sustain delivery of psychological and pharmacological therapy.

**Ethics registration:**

The study was approved by the UCSF Institutional Review Board (IRB), the Kenyatta National Hospital-University of Nairobi Ethics and Research Committee, and the Kenya Pharmacy and Poison’s Board. It was registered on ClinicalTrials.gov (NCT03466346), registration date 2018-03-15, https://clinicaltrials.gov and conducted by Human Subjects Protections (HSP) and Good Clinical Practice (GCP). The trial was monitored by the United States National Institutes for Health (NIH) through PPD.

## INTRODUCTION

Kenya has an estimated 1.9 million depression cases, fifth among African countries after South Africa (2.4 million cases), the Democratic Republic of Congo (2.9 million cases), Ethiopia (4.5 million cases), and Nigeria (7.1 million cases)^1^. A recent household survey in western Kenya revealed that 48% of adults had experienced severe trauma, with a post-traumatic stress disorder (PTSD) prevalence rate estimated at 10.6%, defined as six or more on the trauma screening questionnaire (TSQ)^2^.

In 2021, mental health conditions imposed a significant financial burden on the Kenyan economy, amounting to 62 billion Kenyan Shillings (KES) (Int$ 572 million), 0.6% of the country’s 2020 gross domestic product. These costs can be broken down into two primary categories: healthcare expenditures (5.5 billion KES) and lost productivity (56.6 billion KES), stemming from premature mortality, absenteeism, and presenteeism (lack of focus at work). Such high costs underscore the urgent need for effective interventions, particularly for common conditions^3^.

Public expenditures on mental health are minimal in low and middle-income countries (LMICs) (less than US$ 2 per capita)^4^.The WHO estimates show that governments allocate less than 1% of their health budget to mental health services^5^. In most LMICs, government spending on mental health is vastly insufficient for the public health burden. In Kenya, less than 6% of the country’s GDP is spent on healthcare, against the 15% agreed in the Abuja Declaration, and mental health comprises just 0.01% of total health spending^6^. The Kenya Mental Health Policy 2015–2030 focuses the following priority actions: increasing the budgetary allocation to mental health services to a minimum of the recommended WHO standards both at national and county health sector budgets and ensuring that the health insurance system does not discriminate against persons with Mental, Neurological and Substance use (MNS) disorders in accessing insurance policies^7^. However, the budget allocation has not been achieved as of 2025.

Delivering mental health services integrated with primary care is recommended by the World Health Organization^8^. Compared to participants who receive traditional non-integrated mental health care, those who receive integrated mental health services have a 20% lower risk of hospitalization, a 15% increase of medication adherence and an 85% higher satisfaction rate^9^. Treatment options like Interpersonal Psychotherapy (IPT) and fluoxetine (FLX) are effective for depression and PTSD, with IPT focusing on improving interpersonal relationships and fluoxetine enhancing serotonin levels^10^.

Economic evaluations are an important tool within the priority setting process, whereby decision-makers allocate resources between existing and/or new healthcare services^11^. Understanding the economic benefits of mental health interventions can lead to optimized strategies for integrating mental health care into primary healthcare systems. The use of a Cost Benefit Analysis (CBA) framework for policy appraisal is likely to be particularly useful for assessing gains vs. coss, and for allocating resources across sectors, especially when trade-offs need to be made and appraisals across sectors need to be consistent and comparable^11,12^.

By ensuring that mental health services are adequately funded and supported, providers can enhance treatment, accessibility, and quality, contributing to a more resilient healthcare system. In a resource-constrained environment, such informed decision-making is essential for improving both individual and societal well-being. Toward that goal, this study contributes a cost-benefit analysis of IPT and FLX for treating depression and PTSD in primary care settings in Kenya.

## METHODS

### Study Design

The SMART-DAPPER project in western Kenya was a Sequential, Multiple Assignment Randomized Trial (SMART) that trained and deployed a non-specialist workforce to provide IPT and clinicians (nurses and clinical officers) without an additional mental health specialization, to prescribe FLX for depression and PTSD for adult public sector primary care participants. Fluoxetine, a selective serotonin reuptake inhibitor (SSRI), is on the WHO list of essential medications for Kenya and is available nationwide to public sector healthcare facilities from Level 4 (Sub-county/District Hospitals), Level 5 (County Referral Hospitals), and Level 6 (National Referral Hospitals), through the Kenya Medical Supplies Authority (KEMSA). IPT has been previously adapted and tested in the region and shows strong efficacy^10^. Due to health safety and travel restrictions created by the COVID-19 pandemic, study participants had the option of receiving some of the treatment sessions in-person or via audio-only mobile phone (12).

### Study Setting and study participants

The data were collected between September 2020 and July 2022. Participants were recruited from the general outpatient primary care services at the Kisumu County Referral Hospital (KCRH) and other nearby public health facilities, were included if they screened positive for Major Depression Episode (MDE) and/or PTSD on the Mini-International Neuropsychiatric Interview (MINI), were 18 years of age or older, and could attend weekly IPT sessions/FLX monitoring appointments. Participants were excluded from the study if they met any of the following criteria:

- **Cognitive Dysfunction:** Individuals with cognitive impairments that hindered their ability to participate in or accurately take FLX, such as lack of orientation to person, place, time, or situation.
- **Acute Suicidality:** Those with a moderate or high score on the MINI suicidality module, indicating a need for a higher level of care.
- **Substance Use Disorders:** Participants with drug or alcohol use disorders requiring treatment, as indicated by an AUDIT score of 8 or higher or a DAST score of 3 or higher.
- **History of Mania:** Individuals with a history of mania or those requiring treatment for hypomania, as indicated by a positive score on the MINI mania/hypomania module.
- **Pregnancy or Breastfeeding:** Individuals who were pregnant or breastfeeding.
- **Concurrent Mental Health Treatment:** Participants receiving outside mental health treatment during the study phases were excluded. While any mental health treatment was permitted during follow-up phases (which were recorded by the study team), participants were asked not to initiate new mental health treatments during the treatment period. Those who did were classified as dropouts.

### Overview of Cost-Benefit Analysis

A CBA is a comparative analysis of all costs to all benefits of a program or intervention, where all costs and all benefits are measured in monetary terms^13^. It helps to rank and prioritize options considering their costs and benefits to society. Options with a benefit: cost ratio greater than one or benefits exceeding costs are desirable, more so with high ratios or large differences. A CBA can assist in communicating and justifying investment decisions to stakeholders, such as researchers, policy makers, and citizens^14^ The benefits: cost ratio is then estimated by dividing the benefits by the costs.

### Data collection

The costing for IPT and FLX treatments in primary care settings was done using micro-costing, which is a cost estimation method employing detailed resource utilization and unit cost data to generate an estimate of economic costs^15^. This process began by collecting data on key resources used for IPT and FLX treatment, including session duration, the number of sessions per treatment course, and the monthly salary of treatment providers.

For both IPT and FLX, we calculated the total hours per treatment course by multiplying the session duration in minutes by the number of sessions. The core provider costs for IPT and FLX were derived from the Ministry of Health’s monthly salary, including benefits of the treatment providers, adjusted according to the visit time allocated for each therapy type. Additional costs were included in the analysis, such as the cost of screening participants and supervision, which was set at 10% of core provider costs.

Medication costs were also estimated. The number of fluoxetine pills prescribed was recorded, along with the associated cost per pill, which was 5 KES based on government procurement records. The total cost per individual receiving FLX was determined by summing the core provider costs, screening costs, supervision fees, and medication expenses. Finally, for IPT and FLX, we estimated the total cost per individual in Kenyan Shillings (KES) and converted to USD. Detail in the Excel supplement.

For productivity data, the study used a questionnaire adapted from the World Bank **Living Standards Measurement Study (LSMS)**^16^. These results were reported, currently in a pre-print^17^. In brief, LSMS surveys collect data on economic and health dimensions of household well-being, including consumption and income. We examined repeated measures: baseline and follow-up at 3, 6, and 9 months. We calculated the benefits to individuals associated with treatment, both separately for IPT and FLX, and pooled. Specific productivity outcomes were the proportion of participants receiving a monthly income and their mean income among earners. Income was based on wages from work done for a non-household member, agricultural jobs, business, and self-employment in the past month. Productivity gains in the informal and agricultural sectors were converted to monetary values using earnings rates in the local labor market. All currency measurements were in KES, converted to USD rate of 118.02 KES per USD. We derived a monthly mean income measure for each treatment arm by summing the product of percent earning an income and mean income among earners.

### Cost-Benefit Analysis

For the CBA, we compared intervention implementation costs to income. Income was based on wages from work done for a non-household member, agricultural jobs, business, and self-employment in the past month. Productivity gains in the informal and agricultural sectors were converted to monetary values. Baseline data characterized the economic status of participants before receiving treatment. At the end of the first line of treatment, the same metrics were assessed to determine changes. The analysis calculated the percentage of participants earning income following treatment, along with the mean monthly income among those earners. The difference between the income metrics before and after treatment was then identified, reflecting the gains attributed to IPT and FLX. Income gains per month were translated to annual and 10-year totals by multiplication and discounting to the present, conservatively ignoring study evidence suggesting rising income among individuals with successful clinical outcomes. The benefit-cost ratio for the first year was the change in income divided by intervention cost. This ratio compared the financial benefits derived from increased income post-treatment with the costs for treatment.

For the ten-year analysis, we assumed that clinical relapse would decrease productivity gains proportionally. We searched for and could not find evidence of these relapse rates in similar settings. Thus, we examined a wide range of potential annual relapse rates: 10%, 25%, and 50%. We adjusted for projected wage growth and inflation, and discounted at 3% annually. Detail in Excel supplement.

### Ethics

The study adhered to the ethical principles outlined in the Declaration of Helsinki. The trial was approved by the UCSF Institutional Review Board (IRB), the Kenyatta National Hospital-University of Nairobi Ethics and Research Committee, and the Kenya Pharmacy and Poison’s Board. It was registered on ClinicalTrials.gov (NCT03466346), registration date 2018-03-15, https://clinicaltrials.gov and conducted by Human Subjects Protections (HSP) and Good Clinical Practice (GCP). The trial was monitored by the United States National Institutes for Health (NIH) through PPD. This study adhered to CONSORT guidelines.

## RESULTS

### Treatment Implementation

This study had a sample size of 1918, with 986 participants in the IPT arm and 932 in FLX. Initial remitters included 782 undergoing IPT and 798 receiving FLX. 104 participants transitioned from IPT to FLX and 100 from IPT to a combination of IPT and FLX. These figures were extracted from data for visits for each phase of the treatment. Of those initially receiving FLX, 71 switched to IPT, and 63 to IPT and FLX. In terms of session duration, IPT typically involves longer sessions (60 minutes) compared to FLX (20 minutes). Participants undergoing IPT had a mean of 11.5 sessions per treatment course; those on FLX had 5.3 sessions. This resulted in higher total treatment time for IPT (11.5 hours) compared to FLX (1.8 hours).

### Cost results

The study compared the costs associated with IPT and FLX. A total of 986 participants received IPT, while 932 received FLX. The total cost per individual for IPT was 5,050 KES ($42.79 at prevailing currency exchange rates) compared to 2,511 KES ($21.28) for FLX. (Table 1). The core provider costs per full treatment varied, with IPT at 3,915 KES compared to 1,060 KES for FLX. Additional costs, such as screening and supervision, were accounted for. Medication costs were substantial for FLX, with an average of 140 pills prescribed, costing 700 KES per individual.

### Productivity results

Both IPT and FLX participants reported increased income. Accounting for both the likelihood of earning an income, and the amount of that income, individuals treated with IPT gained KES 13,239 and FLX gained KES 16,242 in year 1. IPT remitters reported a larger gain in income-earning and greater reductions in absenteeism and presenteeism than did non-remitters. More details on the productivity benefits have been published as a pre-print^18^.

### Cost-benefit analysis

Overall, both treatments provided net economic benefits, with FLX demonstrating a higher benefit-cost ratio than for IPT (Table 2). During the first year, for IPT, the percentage of participants earning income increased from 54.9–59.8%, with an annual income among earners rising from KES 50,280 to KES 73,320, resulting in a per-participant income gain of KES 16,242 against a treatment cost of KES 5,050, yielding a benefit-to-cost ratio of 3.2:1. FLX showed a percentage increase from 0.545 to 0.615, with annual earnings growing from KES 46,800 to KES 63,000, leading to a per-patient income gain of KES 13,239 versus a treatment cost of KES 2,511, resulting in a benefit-to-cost ratio of 5.3:1.

### Sensitivity Analysis

The sensitivity analysis evaluated the benefit-cost ratios of IPT and FLX treatments for depression and PTSD over ten years, varying annual relapse rates (Table 3). For IPT, the income gains adjusted for inflation were KES 91,608 with a benefit: cost ratio of 18.1 at a 10% relapse rate, KES 55,215 with a benefit: cost ratio of 10.9 at 25%, and KES 30,539 with a benefit: cost ratio of 6.1 at 50%. In contrast, FLX showed income gains of KES 76,966 and a benefit: cost ratio of 30.7 at a 10% relapse rate, KES 45,908 with a benefit: cost ratio of 18.3 at 25%, and KES 25,051 with a benefit: cost ratio of 9.98 at 50%. Thus, FLX had higher benefit: cost ratios across all relapse rates. Details are available in an Excel supplement.

## DISCUSSION

We compared delivery costs to increases in economic productivity for two primary care-based treatments for depression and PTSD in Kenya, finding that economic gains exceed implementation costs by ratios of 3:2 to 5:2 in the first year. These benefitcost ratios are projected to increase to as high as 30.05 over ten years, depending on the therapy and assumptions about relapse rates. Thus, the potential benefits of mental health interventions like IPT and FLX for treating depression and PTSD are evident, of notably importance in resource-limited settings such as Kisumu, Kenya. Both treatments offer impressive clinical outcomes, making them valuable options for improving mental health care. Furthermore, the economic gains reported by both treatment groups highlight the potential for mental health interventions to contribute to increased productivity in the workforce. This has broader implications for economic development, particularly in low-resource settings where mental health issues are often stigmatized and underfunded. By demonstrating the tangible financial benefits of effective treatment, this study could serve as a catalyst for increased investment in mental health services.

For mental health care in Kenya more generally, benefit-to-cost ratios vary due to the diverse components involved in implementation, which presents important considerations for policymakers and healthcare providers. The Kenya Mental Health Investment case shows that while investing in depression interventions generates economic benefits, the benefits-to-cost ratio is relatively modest at 2.2:1 at 20 years and 1.8:1 at 10 years. This difference from our results can be attributed to several factors, including the costs associated with inpatient care for individuals with severe mental health conditions, the frequent need for outpatient visits for effective management, the ongoing expenses of essential psychotropic medications, and the broader program costs encompassing management, administration, and training of healthcare providers^19^. In addition, our trial focused on comparing real-world treatment strategies suitable for non-specialist primary care, with no untreated controls. Thus, we could not adjust for spontaneous remission, which would likely have decreased the productivity gains associated with treatment.

In our analysis, starting treatment with FLX demonstrated a more favorable benefit-to-cost ratio than for IPT, especially in the sensitivity analysis, which indicates that it may be more sustainable in the long term. This raises a pivotal question about balancing cost-effectiveness with treatment intensity (18)(19). Our sensitivity analysis, which revealed that initial FLX consistently demonstrated higher benefit-to-cost ratios compared to initial IPT across different relapse rates, supports this perspective. This finding suggests that while IPT may offer in-depth treatment, FLX is a more sustainable option for scaling interventions within the Kenyan healthcare system.

A key limitation of this study is the failure to consider government’s commitment to investing in mental health. While the cost-benefit analysis focused on costs linked to income increases following treatment with IPT and FLX, it did not account for the individuals’ perceived value of these interventions. Incorporating willingness to pay for these health benefits could provide a more comprehensive understanding of the overall value of mental health interventions in primary care settings.

## CONCLUSION

In conclusion, while both IPT and FLX have their merits, and importantly both works clinically and economically, the choice between them may consider economic viability and patient preference. Future studies should explore long-term outcomes and the implications of treatment transitions, as well as the integration of mental health services into primary care, to enhance accessibility and effectiveness in diverse populations.

## Supplementary Files

This is a list of supplementary files associated with this preprint. Click to download.

- CONSORTchecklist9thJuly2025.docx

## Figures and Tables

**Table 1:** Cost of IPT and FLX treatment, Primary Care, Kisumu, Kenya

	Overall cost by Arm	
	IPT	FLX
First Line Therapy (Remitters)	(N = 782)	(N = 798)
Session duration (minutes)	60	20
Sessions per treatment course	11.5	5.3
Hours per treatment course	**11.5**	**1.8**
Monthly salary for treatment provider (KES)	51,070	90,000
Core provider costs per full treatment (KES)	3915	1060
Cost of screening (KES)	150	150
Supervision at 10% (KES)	392	106
Medication # pills (20 mg)	-	140
Medication cost (5 KES)	-	700
Cost per individual (KES)	4,457	2,066
**Full Cohort** *	N = 986	N = 932
Cost per individual (KES)	5,050	2,511
Cost per individual (USD)	$42.79	$21.28

* Full details of costs for individuals requiring further treatment is provided in the Excel supplement.

**Table 2:** Cost-benefit analysis for IPT and FLX (Year 1), Treatment of Depression & PTSD, Primary Care, Kisumu, Kenya

	Before Treatment			End of First Line Treatment			Difference (Gain)	Treatment cost	Benefit: Cost Ratio
Initial Therapy	% earning income (prior month)	Annual income among earners (mean, KES)	Annual income among all participants (mean, KES)	% earning income (prior month)	Annual income among earners (mean, KES)	Annual income among all participants (mean, KES)	Annual income among all participants (mean. KES)	Across all treatment paths (KES)	
IPT	54.9%	50,280	27,604	0.598	73,320	43,845	16,242	5,050	3.2
FLX	54.5%	46,800	25,506	0.615	63,000	38,745	13,239	2,511	5.3

**Table 3:** Cost-benefit analysis for IPT and FLX (Ten Years, Varied Relapse Rates), Treatment of Depression & PTSD, Primary Care, Kisumu, Kenya

	Annual Relapse 10%		Annual Relapse 25%		Annual Relapse 50%	
	Income Gain (KES) *	Benefit: Cost Ratio	Income Gain (KES)	Benefit: Cost Ratio	Income Gain (KES)	Benefit: Cost Ratio
IPT	91,608	18.14	55,215	10.93	30,539	6.05
FLX	76,966	30.65	45,908	18.28	25,051	9.98

## Abbreviations

CBA: Cost-Benefit Analysis
FLX: Fluoxetine
GCP: Good Clinical Practice
GDP: Gross Domestic Product
HSP: Human Subjects Protections
IPT: Interpersonal Psychotherapy
IRB: Institutional Review Board
KEMRI: Kenya Medical Research Institute
KEMSA: Kenya Medical Supplies Authority
KES: Kenya Shillings
LMICs: Low and Middle-Income Countries
LSMS: Living Standards Measurement Study
MDE: Major Depression Episode
MINI: Mini-International Neuropsychiatric Interview
MNS: Mental, Neurological and Substance use
NIH: National Institutes for Health
PTSD: Post-Traumatic Stress Disorder
SMART: Sequential, Multiple Assignment Randomized Trial
SSRI: Selective Serotonin Reuptake Inhibitor
TSQ: Trauma Screening Questionnaire
UCSF: University of California, San Francisco
USD: United States Dollar
WHO: World Health Organization
